# Systems biology and brain activity in neuronal pathways by smart device and advanced signal processing

**DOI:** 10.3389/fgene.2014.00253

**Published:** 2014-08-26

**Authors:** Gastone Castellani, Nathan Intrator, Daniel Remondini

**Affiliations:** ^1^Department of Physics and Astronomy, L. Galvani Center for Biocomplexity, Biophysics and Systems Biology, University of BolognaBologna, Italy; ^2^Department of Computer Science, Exact Sciences Faculty, Tel Aviv UniversityTel Aviv, Israel

**Keywords:** multivariate analysis, multiple networks, electroencephalography, genomics, metagenomics

## Abstract

Contemporary biomedicine is producing large amount of data, especially within the fields of “omic” sciences. Nevertheless, other fields, such as neuroscience, are producing similar amount of data by using non-invasive techniques such as imaging, functional magnetic resonance and electroencephalography. Nowadays a big challenge and a new research horizon for Systems Biology is to develop methods to integrate and model this data in an unifying framework capable to disentangle this amazing complexity. In this paper we show how methods from genomic data analysis can be applied to brain data. In particular the concept of pathways, networks and multiplex are discussed. These methods can lead to a clear distinction of various regimes of brain activity. Moreover, this method could be the basis for a Systems Biology analysis of brain data and for the integration of these data in a multivariate and multidimensional framework. The feasibility of this integration is strongly dependent from the feature extraction method used. In our case we used an “alphabet” derived from a multi-resolution analysis that is capable to capture the most relevant information from these complex signals.

## INTRODUCTION

Brain activity is without doubt the most complex process in nature. While the body of research is exponentially growing, it is quite amazing that fundamental building blocks or atoms of this process are still quite unknown. Two of them indicate how far we are in understanding brain processes; the first is the fundamental synaptic modification rule in a single neuron, and the second is internal brain representations of the physical world (and sensory input).

For a long time, it was assumed that it would be possible to describe the synaptic modification rule by deducing from observations, and analyzing them mathematically (Lynch et al., 1990; Cooper et al., 2004) in a similar way as other physical rules have been discovered. As the process turns out to be extremely complex in terms of the different neuro-transmitters, neuro-receptors and the chemical interactions which lead to the changes, it is now assumed that further deductions and a potential breakthrough in understanding synaptic modification may be obtained by massive computer simulations (Kandel et al., 2013). This is motivated by the immense progress computers have made in the last two decades, and the believe that computational power and memory which resembles the brain will be reached in a decade (Kurzweil and Grossman, 2005).

The quest for understanding the internal brain representation is somewhat independent of the quest for understanding synaptic plasticity. To illustrate how little we know about internal representations, we can take an object such as a desk, and point out that we do not know what it is that makes the simple combination of a surface and legs be represented (or recognized) as a desk. Specifically, what is the difference in representation for two (similar desks), is it mainly temporal, namely a different form of oscillation of the same neurons, or spatial, mainly activity of different neurons (Biederman, 1987; Edelman, 1999).

This somewhat frustrating description of the current state of the art suggests that a certain change in the way we collect data about the brain may be necessary so as to drive us to more meaningful conclusions.

A step in that direction occurred when functional MRI (fMRI) became popular. Then, not only we moved away from determining brain representations, but we also started looking at brain activity in a very crude way. Looking at oxygenated blood to different regions of the brain as a marker for neural activity in those regions, and doing so while integrating data in 3 s time windows. This crude brain activity measure led to great progress in brain activity interpretation and in attributing functional labels to different brain regions. Then came an even more surprising finding; we realized that we do not need to fully understand the role of certain regions in various cognitive and emotional tasks. Instead, it is enough to know the typical (crude) pattern of activity in a group of normal people, and apparently, an attempt to alter the activity in such regions in a group of subjects that suffers from some brain malfunction, may alleviate symptoms of that malfunction.

This paper suggests that another step forward in understanding brain activity and improving brain malfunction may come from developing new methods which like fMRI, provide a view on different functional units of the brain, but, unlike fMRI can be taken outside of the clinical setup and put into continuous mobile use to operate in any environment and thus enrich our ability to observe brain activity under natural settings.

To motivate this, we note, that it is remarkable how much we have learned about brain networks of activity from fMRI given its temporal and clinical limitations (Cabeza and Nyberg, 2000).

The electroencephalography (EEG) is a much older method for sensing non-invasively the functioning brain, with human recordings starting in 1924 (Haas, 2003). The electrical activity mainly results from fluctuations in ionic current flows within (1000 or more) neurons and it provides an indication to the type and degree of activation of different brain regions (Niedermeyer and Lopes da Silva, 2005). Throughout the century of EEG research, EEG energy features were extracted from a small number of frequency bands (e.g., Klimesch, 1999) and other features were extracted from time-locked averaging (ERP and EP) of the response (for review see: Luck, 2005). As the role of EEG in characterizing epilepsy was discovered, it was determined that epilepsy is some form of excessive synchrony between neurons and between brain regions. This has led to the discovery of more advanced signal processing methods which are sensitive to early synchrony changes (Fisher et al., 2005). However, more advanced signal decomposition and feature extraction methods have emerged only very recently in the analysis of EEG data (Duncan et al., 2013; Intrator, 2014).

It is likely that in the near future, there will be several new brain activity representations, all of which will be rich in content and will provide orders of magnitude more data as they will enable continuous mobile monitoring. This paper discusses the usage of such advanced methods, and application of methods which were mainly developed for genomic data analysis, in brain activity interpretation.

There is indeed, a huge overlapping between methods used in genomic data analysis and methods used for brain-activity interpretations. Among the most used we can quote correlation methods, that has been used both for large scale gene-network analysis and for several brain data analysis and modeling (Cooper et al., 2004; Remondini et al., 2005). Other overlapping between these two fields are given by the role of noise in the spontaneous background activity in neural and genomic systems and the subsequent modeling strategies (Milanesi et al., 2009) mutuated from the field of complex systems. In the last 20 years another unifying concept has been developed within the field of statistical mechanics and complex systems: the concept of complex network (Albert and Barabási, 2002). The idea of complex network has been applied to neural systems and to genetic systems by the fundamental tool of connectivity and degree distributions such as the famous power law that is observed in both systems. As a further analogy, at least from the point of view of modeling and data analysis, there is the concept of pathway. The pathways analysis for genomic systems is now a common tool that provide a better interpretation and simplification of this complex data (Francesconi et al., 2008). Nevertheless, the neuronal pathways, or neuronal circuits and areas, have a long history in neuroscience, starting from the classical phrenological idea, about the localization of emotions and neuronal functions. The modern imaging tools and methods are now supporting and confirming the fact that neuronal functions are precisely localized in the brain and that there is a strong relation between the anatomical and the functional localization. This is exactly the same that is observed in cells and tissues by pathways analysis.

In this paper we will take in exam the relations between the genomic and neuronal data analysis and modeling and will illustrate how this can be a powerful method for the analysis of a new generation of data obtained from EEG. We strongly believe that this method will be a further advancement in the field of Systems Biology.

## NOVEL BRAIN ACTIVITY INTERPRETATION

Electroencephalography sensing started at the beginning of the 20th century (see Swartz, 1998 for a full review). The first recording of EEG from humans occurred in 1923, with the seminal work of Hans Berger (Haas, 2003), who discovered the Alpha and Beta rhythms of brain-wave oscillations. Later, other typical oscillations were discovered; those below alpha and those above beta. With multi-electrode recording, it became apparent that the EEG signal is not uniform across the skull, and that the signal observed in each electrode is strongly affected by the cortical volume closest to that electrode. This enabled the analysis of correlations of signals between different regions (electrodes), or as is thought now, between different (distributed) cortical networks (Buzsáki and Draguhn, 2004).

While EEG is not considered spatially accurate, the analysis of activity correlations across electrodes gave research a strong boost, in particular, it enabled de-correlating between different sources of brain activity using blind source separation methods such as independent components analysis (ICA; Delorme and Makeig, 2004). The introduction of ICA tools to the EEG community which was mainly done by Delorme and Makeig (2004), led to a large body of work in the analysis of EEG under many brain state conditions. It also enabled an efficient artifact removal (mainly due to muscle activity) from EEG data.

From this short review, one can conclude that separation or decomposition of the EEG signal into different components is a very effective way to study different brain networks in separation. The question becomes, whether an electrode array is essential for such separation.

While the body of work on multi-channel EEG signal decomposition is huge, the amount of work on single-channel EEG decomposition is very small. It was used for example to adapt the features to different subjects for brain computer interface, but from a 32-electrode cap (Yang et al., 2007). In this paper, we concentrate on EEG signal decomposition from a single EEG lead which is given as the difference of two EEG electrodes. The signal difference between two frontal EEG electrodes can provide the simplest measure of Cerebral Asymmetry (Henriques and Davidson, 1990). This asymmetry has long been associated with emotional reaction as well as during cognitive tasks (Davidson, 1988). Thus, if one wants to select a single EEG lead that can cover bot emotional and cognitive brain states, it makes sense to use the difference between Fp1 and Fp2, which are two frontal electrodes.

Luckily, these electrodes reside on the forehead and thus, may be easier to put, and can be dry without the need of a conductive gel.

**FIGURE 1:**
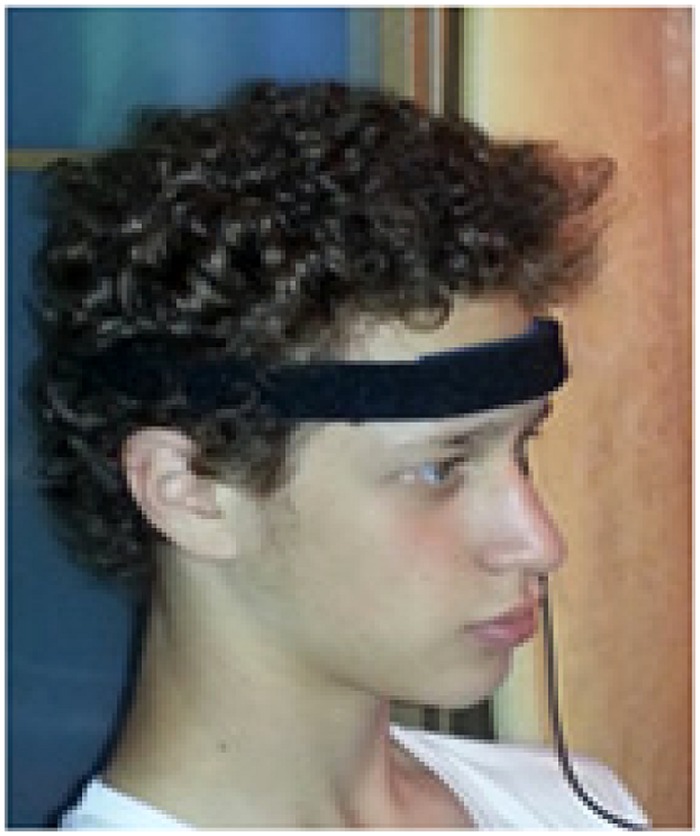
**The EEG sensor**.

Using a 3-sensor EEG as in **Figure 1**, Intrator (2014) has discovered features that can be obtained from a single EEG lead and may be useful for emotional and cognitive brain state discovery. These were found using a two stage process: first, a signal processing and decomposition is applied to propose candidate features, and then, big-data mining and robust statistics methods are used to prune the features and test the robustness and universality of the remaining features across subjects and across conditions. These brain activity features (BAF) provide potential new insights on brain activity and states. They distinguish between three major types of activity: focused, distributed, and chaotic.

**FIGURE 2:**
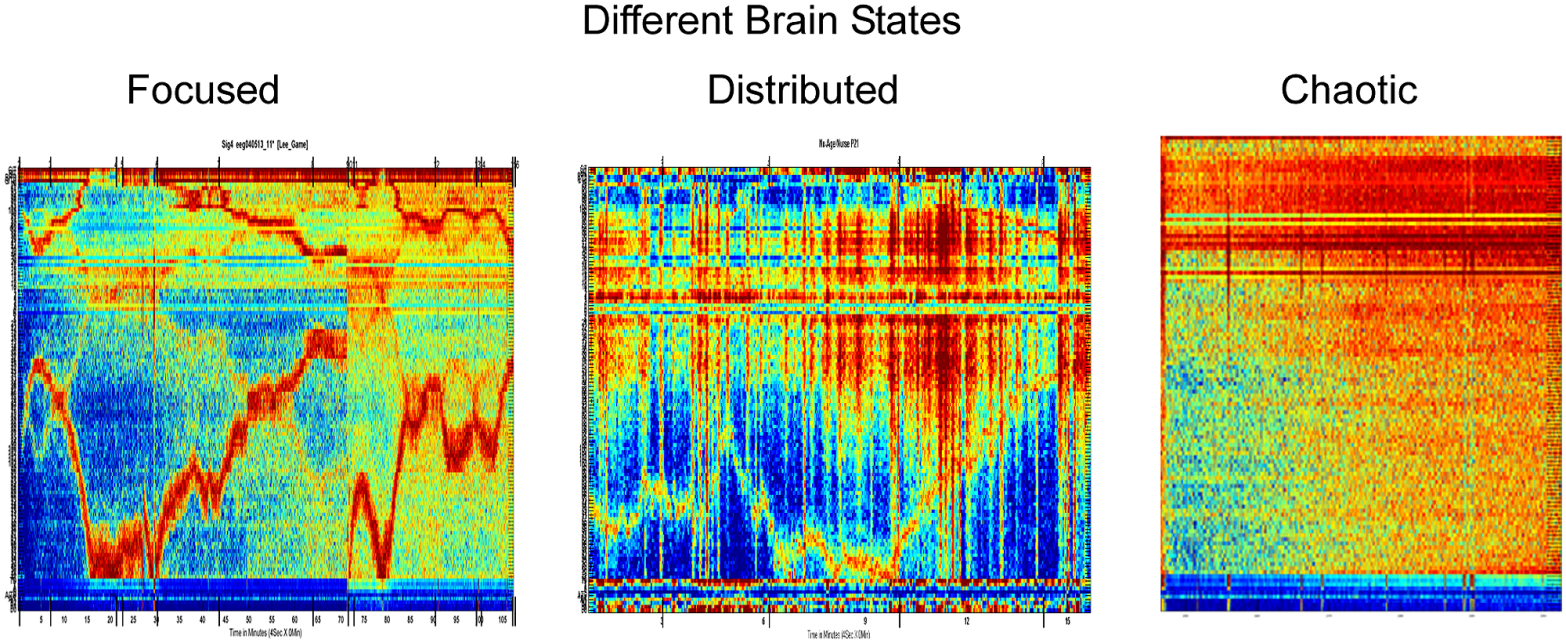
**Three different brain states based on the brain activity features found by Intrator (2014)**.

**FIGURE 3:**
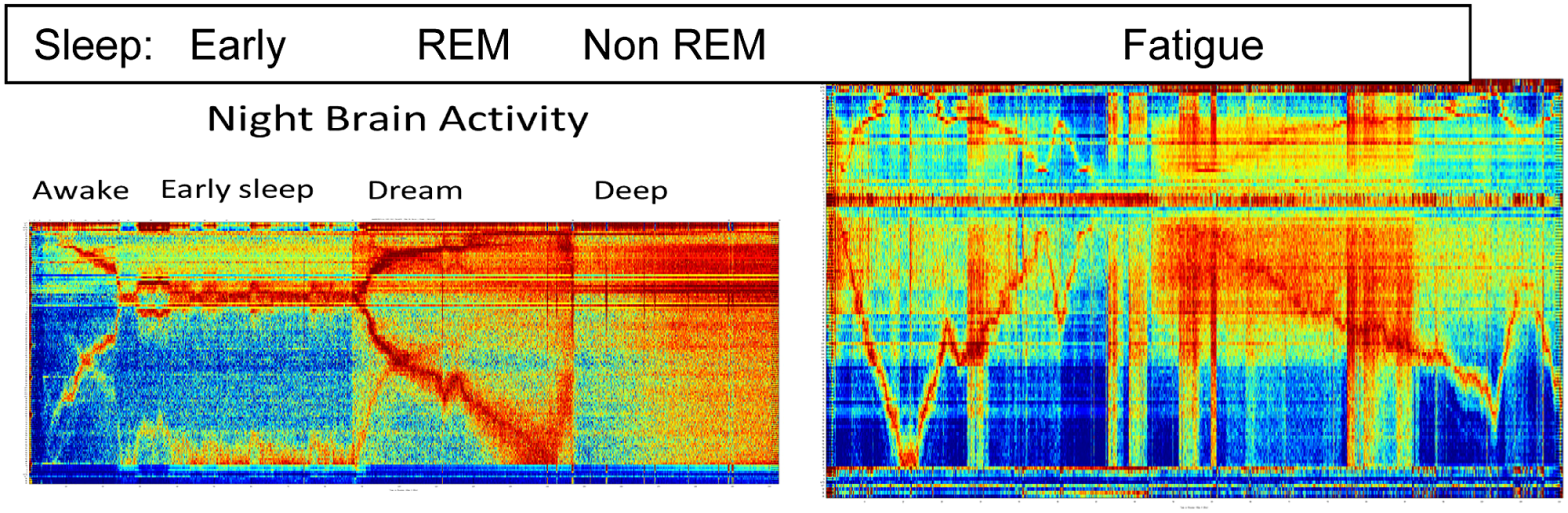
**Different brain activities features during sleep and Fatigue.** See text for details.

Before describing the distinction, we briefly explain what can be seen in **Figures 2** and **3**. Each column of each panel represents the activity of a single BAF (in this case, 121 different features) at a certain consecutive time point of about 1 s. In all panels, the BAFs are the same and are ordered in the same order. Each panel represents about an hour of brain activity. The BAFs which were obtained from different subjects, use the heat color map is used to represent the magnitude of activity, so the more brown/red each pixel is the more active the corresponding feature in the specific time location is. From the activity during the “focused” state, it is apparent that there is a certain correlation and continuity between the features, so that the activity, which can change in time between different features, changes in a continuous way, so that features that are presented close to one another are more likely to become active. The chaotic stage of non-REM sleep is the only exception.

The relation between these features and well-known EEG features or known areas and networks of brain activity is subject to study and will be described elsewhere. Some indications from anecdotal evidence suggest that the activity in the early part of sleep resembles activity during Anesthesia and during some forms of meditation. From studies done on that meditation performed during fMRI scans, we deduce that these specific features correspond to activity in the medial pre-frontal cortex.

**Figure 3** depicts the richness of the brain states as is observed by the BAF during sleep and fatigue.

The left panel represents close to 3 h of activity while the right panel represents about an hour and a half of activity. Clear distinction between three known sleep stage are see and they correspond to the early, REM and non-REM stages.

As is well known, sleep monitoring is crucial for the early detection of physical and mental health problems; diagnosis and treatment of insomnia; and diagnosis and monitoring of dementia. Fatigue monitoring is crucial when the brain is engaged in tasks that require fast thinking and response, especially in roles where alertness is essential to performance and safety (e.g., a pilot). The right panel indicates the strength of the BAF for fatigue monitoring: it depicts the brain activity of a subject briefly falling asleep while watching a movie. Temporal regions where stronger and weaker engagement with the movie are clearly visible, as well as the length and depth of sleep.

## COMPLEX NETWORK THEORY

In the last decade, physics has been expanding to new research areas. In particular, life-related sciences (ecology, sociology, economics, and last but not least biology) have been showing striking analogies with complex systems arising from various physical areas. Such approaching has happened from both fronts: on the life science side, huge amounts of data have become available for detailed analysis, thanks also to the Internet, through which this data is nowadays easily collectable and queryable (e.g., stock market financial series, social networks, high-throughput biological data). On the other side, many physical and mathematical tools, that had been proven useful in explaining complex phenomena like polymer growth or spin glass, began to spread to other research areas like biological and social sciences in a broad sense.

The common trait of these research fields can be found in the framework of network theory, which focuses on the relationships among elements and allows to draw general conclusions, even though the details of the system are not completely known or easily tractable from a mathematical point of view. Relaxing the attention to the details of the specific interaction or element, network theory aims to provide tools for the characterization of a set of relationships, represented as edges or *links*, occurring among similar elements, referred to as vertices or *nodes*.

One of the most powerful approaches to physical systems is statistical mechanics. Many results (for “ideal” gases or solids) have been obtained by considering random interactions between elements of the system, so that a “mean field theory” could be built from the average behavior of the system. The main drawback of this mean field approach (and the actual challenge at the same time) is that complex systems (to which living and life-related systems belong) are often characterized by a non-trivial set of interactions, and a mean field approach can completely miss the interactions. Moreover, social and biological systems can be considered as constantly far-from-equilibrium systems, since equilibrium for every life-related process equals to death, and a continuous influx and eﬄux of energy and matter is necessary to maintain life-suitable conditions. It is thus quite hard to fit them into equilibrium-based models that we can say to constitute the “core” of classical statistical mechanics.

An approach that has received renewed attention is based on the so called Master Equation (CME) that describes the temporal evolution of the probability of having a given number of molecules for each chemical species involved. The discrete probabilistic approach, as with CME, is attractive because it ensures the correct physical interpretation of fluctuations in the presence of a small number of reacting elements (as compared to continuum approaches as Langevin and Fokker-Planck formalism; van Kampen, 2007) and because it provides a unitary formulation for many biological processes, from chemical reactions to ion channel kinetics. The CME theory can be related to predictions on the noise levels in selected biological processes, as for example during transcription and translation (Friedman et al., 2006). In particular, the observation that mRNA is produced in bursts varying in size and time has led to the development of new models capable of better explaining the distributions of synthesized products (Cai et al., 2006).

The models based on CME can help to characterize the role of noise in networks reconstruction as well as the role of fluctuation in the enhancement and maintenance of biological functions.

Furthermore, the ME approach, allows to compute all the thermodynamic quantities, including entropy and free energy, with the consequent possibility to characterize the system as a non-equilibrium system if the detailed balance condition is not satisfied.

One of the greatest contributions, which may be given by network theory to the understanding of biological and social systems, is that the network architecture may reflect the dynamical processes that led to it. In a pure statistical-physical fashion, different “universality classes” can be sought for in order to fit the process we are studying, be it the ask-bid mechanism for a stock, the patterns of gene expression or neuronal activation following a stimulus. We remark that the features of a network model are peculiar from a static viewpoint (e.g., the relation between network topology and the evolutionary model that led to it) and from a dynamic viewpoint (e.g., the responses to perturbation, or the noise features of a stochastic dynamics). Recent models of social networks (Holme and Newman, 2006) show that the situation can be even more complicated, with nodes interactions affecting network topology and network topology affecting node interaction dynamics. This is a common paradigm for biological systems at several levels, for genomic, nervous, and immune (for a recent review, see Gross and Blasius, 2008).

## MULTIPLEX NETWORKS

During the last years, a growing interest in the so called multiplex networks has gradually grown within the scientific community. A multiplex network is a topological structure where individual nodes can have links belonging to several layers of networks at the same time. The multiplex, or multivariate network was well known in social sciences at least starting from the seventies (Boorman and Harrison, 1976).

A useful example for pointing out the differences between networks and multiplex is the analogy, from a mathematical-statistical point of view, with univariate and multivariate data.

A univariate variable is identified by single measurements; for example a population survey to estimate the average weight of elderly. Since we are only working with one variable (weight), we would be working with univariate data.

A multivariate variable is identified by multiple measurements for each sampling unit. If for example, in the same population of elderly, we are collecting not only weights, bur also blood pressure, heights, heart rate, etc, we will have 4-uples of values.

In the field of social science and social networks there are many examples of multiplex. In general, each individual node can have different kinds of social ties or relations or transportation systems where each location is connected to another location by different types of transport.

In social sciences a multiplex is defined on the basis of the existence of multiple relations among actors, where actors are defined accordingly to the actor–network theory (ANT; Latour, 1987; Law and Hassard, 1999). At a larger scale relations among nations are characterized by a plethora of cultural, economic, and political exchanges as well as from other form of connections.

Single networks have been studied extensively (Albert and Barabási, 2002; Boccaletti et al., 2006) also from a dynamical point of view (Dorogovtsev et al., 2008) and in social sciences (Wasserman and Faust, 1994). Nevertheless, in nature there exist many systems that cannot be considered as single networks. Noticeable examples are: transportation networks, climatic systems, economic markets, energy-supply networks, ecological networks, human brain and metagenomic systems (Bianconi, 2013).

Multiplexity is thought to play an important role in the organization of large-scale networks. For example, the existence of different link types between agents explains the overlap of community structures observed in ecological, genomic, metagenomic, and social networks (Szell et al., 2010).

The concept of multiplex is taking new space in modern Biology. As a paradigmatic example we will consider metagenomic data and suitable methods for multivariate associations between multiple set of omic data on the same population.

The human metagenome is the set of *Homo sapien*s genes plus the trillions of genes in the genomes of microbes that live in the human body. The microbial genome (microbiome) is in a dynamical relation with the human organism and helps it by crucial functions such as metabolic processes, shaping, control and protective immune (IS) system development, that helped the (co)-evolution of human being and ultimately also the brain development.

With the term Metagenomics, we define the set of omics measurements aimed to quantify the composition and the interactions dynamics between the host and the microbiome. This includes characterization at the level of DNA (metagenome), RNA (meta-transcriptome), protein (meta-proteome), and metabolic network (metabolome), both for the host and the microbiome. Hence, *H. sapiens* is a metaorganism (or super organism) where the different microbiota present in different organs play a major physiological and pathological role.

The interaction between GM and host is personalized, dynamic, bidirectional, history-dependent and is taking place in a multivariate way, by exchange of various molecules: metabolic, genetic, immunitary etc. The dynamic properties of the GM are caused by the fact that GM is a complex ecosystem with a complex dynamics derived by the interactions with components such as the virome (the set of viruses in the human body) the IS and the Neural System. The natural way to characterize the interaction between GM and host is to perform multiple intersection between metagenomic layers an to reconstruct networks and multiplexes.

From this perspective, social systems and biological systems can be seen as a non-linear superposition of complex networks, where nodes represent “actors,” “genes” or metabolites and links capture a variety of different social and biological relationships. Human societies and biological systems can be regarded as large numbers of locally interacting agents, connected by a broad range of relationships based on exchange of molecules or social relations. These relational ties are highly diverse in nature and can represent a variety or relations (friendship, love, communication) or ecological interactions (exchange of nutrients, predator/prey relationship, cooperation, amensalism, or neutrality).

The networks in the different slices are not independent, their shapes are interconnected and reciprocally influenced; one network can act as enhancer or inhibitor on the other.

**FIGURE 4:**
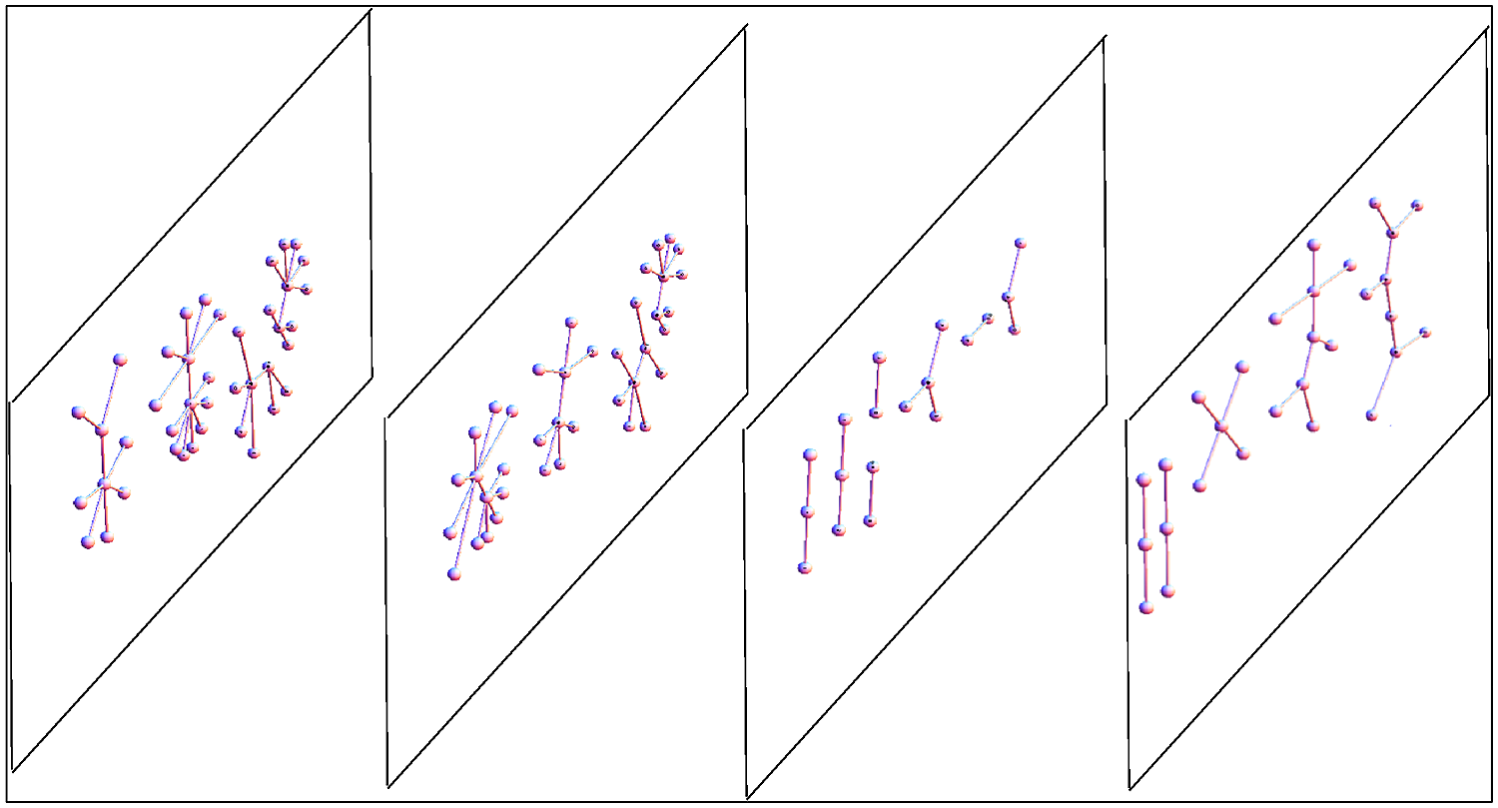
**Scheme of a multiplex network with four layers.** The same nodes appear in every multiplex layer, but every layer can have different internal connections. In general, in every layer we can have different kinds of networks, both in terms of topology or because of different represented relationships. For example, we could have a multiplex in which in one layer there are genes connected by a transcription network, in the second layer the proteins (produced by the genes) can interact, bind, or be co-expressed, and in the third layer the enzymes encoded by the proteins are embedded in a metabolic network. The typical network observables (e.g., connectivity) that in a single network are scalar values for each node, in a multiplex become a vector (one value for each layer), thus the relationship between nodes based on these vectors can be more complex than in a single network.

For instance networks in the brain can have excitatory and inhibitory connections, and these can influence the behavior of neurons in other slices. Another example is the transcriptional network where connections intra-slice can modify connections inter-slice (e.g., splicing and transcription factors). Also the case of metagenomic networks is best understood within the framework of multiplex: the cross-talk between host IS and microbiome is influenced by ecological interactions between the Gut Microbiota. Hence we can say that several biological systems, including the brain, can be characterized as a superposition (a linear combination, or also a non-linear combination) of its networks, all defined on the same set of nodes. This superposition is usually called multiplex, multirelational, multimodal, or multivariate network (see **Figure 4**).

## NETWORK RECONSTRUCTION FROM GENE-EXPRESSION DATA BY *A PRIORI* BIOLOGICAL KNOWLEDGE

High-throughput gene expression analysis has become one of the methods of choice in the exploratory phase of cellular molecular biology and medical research studies. Although microarray technology has improved measurement accuracy, and new statistical algorithms for better signal estimation have been developed (Hekstra et al., 2003; Irizarry et al., 2003; Affymetrix Inc.), reproducibility remains an issue (Fortunel et al., 2003). A way to overcome this difficulty is to extend the analysis, in particular the interpretation of the results, from a single-gene level (in which variablity is maximal) to a higher level in which genes are grouped into functional categories. This approach has been shown to be more robust and reproducible (Subramanian et al., 2005; Manoli et al., 2006), since the “integration” of multiple gene expression patterns may “average out” fluctuations (i.e., false positives). Moreover, it mat lead to an easier biological interpretation of the experimental observations, since the single significant genes are embedded into functional categories or processes of clearer biological meaning.

Gene ontology (GO; Ashburner et al., 2000) and biological pathways are the two main gene-grouping schemes in use. GO organizes genes according to a hierarchy of terms, that from a network point of view is defined as a directed acyclic graph (DAG), in simple terms a “tree” in which genes are the “leafs” and the grouping categories are the “branches” (thus following a hierarchy from the external branches to the “root”). This DAG is divided into three categories: “cellular component,” “biological process,” and “molecular function.” Genes appear in more than one level in each of the three categories, but no relation between genes is described (apart from them being in the same group). The biological pathway database cured by the Kyoto University (Kyoto encyclopedia of genes and genomes, KEGG; Kanehisa and Goto, 2000) is probably the most known: it groups genes into pathways of interacting genes and substrates, and contains specific links between genes and substrates that interact directly. Both databases are manually curated but incomplete, also because the knowledge of gene functions and interactions is still evolving. Each gene belonging to the GO database belongs to several categories, nested as in a phylogenetic tree: starting from a gene, we can reach the root through several branches, representing all the categories it belongs to. A limit of GO is the choice of the categories, that might not be so rigorous or univocal. KEGG provides instead a more detailed organization of the genes, since the relations are the exact biochemical interactions occurring inside the cell, but it contains information on fewer genes than GO, since fewer genes are so clearly characterized in terms of their products and interactions.

Different approaches have been proposed to identify significant gene groups based on lists of differentially expressed genes. Several methods have been implemented that can be directly applied to existing gene-grouping schemes. GOstat (Beissbarth and Speed, 2004) compares the occurrences of each GO term in a given list of genes (tested group) with its occurrence in a reference group (typically all the genes on the array) assigning a *p* value to each term. In the context of pathway analysis, a similar approach is used by Pathway Miner (Pandey et al., 2004) which ranks pathways by p values obtained via a one-sided Fisher exact test. Other methods allow investigators the possibility to define their own gene-grouping schemes. For example, Global Test package (Goeman et al., 2004) applies a generalized linear model to determine if a user-defined group of genes is significantly related to a clinical outcome. With the gene set enrichment analysis (GSEA; Mootha et al., 2003) an investigator can test if the members of a gene set tend to occur toward the top or the bottom of a ranked gene list obtained from the differential expression analysis, and therefore are correlated with the phenotypic class distinction.

In this paper, we extend the significance analysis of gene pathways to higher order structures, i.e., networks of pathways whose intersections contain a significant number of differentially expressed genes. Network structure can reveal the degree of coordination of different biological functions as a consequence of the treatment, as well as the presence of “focal areas” in which groups of genes play central roles. We show examples in which some biological functions (related to specific pathways) are biologically relevant for the studied process, due to their position inside the pathway network. This analysis can be extended to groups of genes at the “interface” between pathways, whose imbalance can affect more than one biological function.

Our approach is aimed at understanding how external perturbations, such as gene activation or tumor induction, can induce in various types of cells, cell lines or derived tissues, behaviors that can generate, integrate, and respond to dynamic informational cues.

The broad question that we are trying to answer is how a cell converts perturbations of its signaling activity into a “binary,” or at least discrete, decision, resulting in the appearance of a given phenotype. Thus the signaling activity has to be diffused within the cell between and within pathways. A signaling pathway is not a rigid unit, since it can achieve one ore more functions with different subsets of its elements. The communication with other pathways, due to the fact that many elements are shared between several pathways, may be captured by looking at those elements belonging to the interface between pathways.

## NETWORKS AND MULTIPLEX FOR BRAIN MODELING AND DATA ANALYSIS

### THE PATHWAY MAPPING

According to the theory of neuronal circuits, a neuronal pathway is formed by a series of interconnected neurons that can be associated with a given response. With this definition, we can use methods for pathway analysis initially designed for gene expression studies and based on network theory (Remondini et al., 2005).

Biological pathways can be identified in two ways:

(1) By *a priori* biological knowledge (supervised method)
(2) By a data driven approach (unsupervised method)

The “*a priori* biological knowledge” approach is based on the idea that we have expert information on pathway structure and interconnections. The classical example is the metabolic and signaling pathways as coded by biochemistry experts (see KEGG, ReconX). In the field of neuroscience this corresponds to relying on the vast literature in brain areas identification based on functional imaging.

The data driven approach, is based on some properties of the collected data. For example, we can define a pathway as a set of neurons (a network) whose activity is associated in time. Correlation with its variants (e.g., parametric and non-parametric) can be used for this purposes. Moreover, it is possible to characterize the causality relationships between data (e.g., brain areas) with several methods. Granger causality (Granger, 1988), is a way to test if a time series X Granger-causes Y, by comparing lagged values of X and Y. It can be used both for searching many-to-one or one-to-one relationships, but for a high-throughput dataset (e.g., fNMR voxel data dynamics) it can be computationally very demanding. Other methods are based on partial correlation (for review Mirowski et al., 2009) and also on the so called Gaussian Graphical Models (Yin and Li, 2012).

Relevance networks (Butte and Kohane, 1999) are a popular method for the analysis of time series of expression levels. The basic idea is to construct a network of similarity of the time patterns. Several similarity measures have been used, such as correlation and mutual information. This technique can represent multiple connections, and capture negative as well as positive correlations. Once the matrix containing the similarity measure for all pairs of genes has been computed, a threshold is used to define the significant links in the network. Network validation can be obtained by permutation testing, i.e., by randomly shuﬄing the time series or just shifting the phase (Schreiber and Schmitz, 2000). A similar approach has been applied to metabolic networks (Martins et al., 2004; Camacho et al., 2005) using computed metabolite correlations to infer changes in regulation using samples from different physiological states.

An alternative approach is offered by graphical Gaussian models (GGM) that use partial correlation as a measure of independence between two genes. Partial correlations are related to the inverse of the correlation matrix, and in GGMs missing edges indicate conditional independence. One of the biggest problems with GGMs is that the correlation matrix is usually singular and cannot be inverted. Different approaches have been proposed to circumvent this problem: restrict the number of elements analyzed to less than the number of samples (Kishino and Waddell, 2000; Waddell and Kishino, 2000; Toh and Horimoto, 2002) use partial correlation coefficients of limited order (de la Fuente et al., 2004; Magwene and Kim, 2004; Wille et al., 2004); approach the matrix inversion as an ill-posed inverse problem through regularization methods (usually via empirical Bayes, such as variance reduction, see Dobra et al., 2004; Schafer and Strimmer, 2005).

Although co-expression is not a direct indication of co-regulation, and it is neither capable to give informations about causal relationship due to its intrinsic symmetry, it is a very useful tool that can be used to interpret the effect of a perturbation in eliciting different phenotypes when combined with an ontology analysis. Moreover, in a time-series correlation-based approach, the choice of the time window can be critical. Most of the state-of-the art analysis (e.g., for defining functional areas in the brain) are based on whole time-series analysis (one long time window) but recent works seem to show that useful information can be extracted also at shorter time scales (Liu and Duyn, 2013). The key point is to assess if the time resolution available by fMRI is enough for these purposes: some simulation works seem indeed to point in this direction, thus justifying the use of small time windows (Honey et al., 2007). The choice of optimal time window size, besides depending on the time resolution of the experimental setup (fMRI and EEG are very different from this point of view), also depends on the characteristic time scales involved in the brain activity process. This also remains an open issue, even if many experimental observations (Buzsáki and Draguhn, 2004) and theoretical models (Haimovici et al., 2013) show a sort of chaotic, or anyway multiscale on a broad range, spectrum of time scales related to brain activity.

**FIGURE 5:**
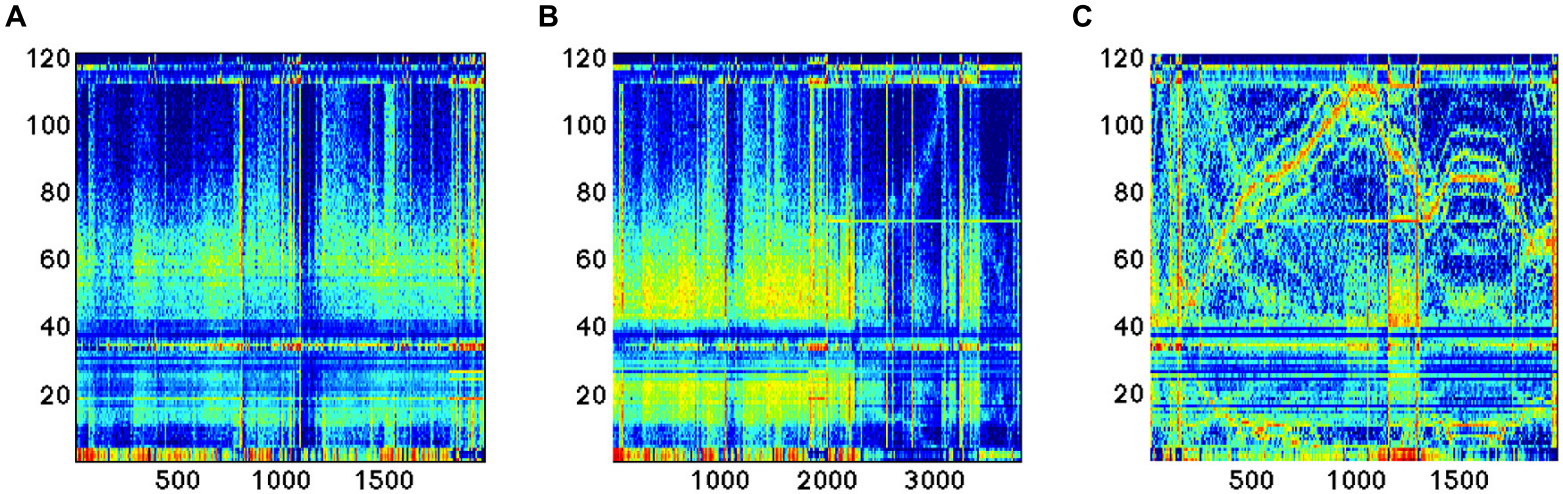
**Time series of the 121 features analyzed during EEG recording in three different conditions: (A,B) sleep; (C) dream activity**.

**FIGURE 6:**
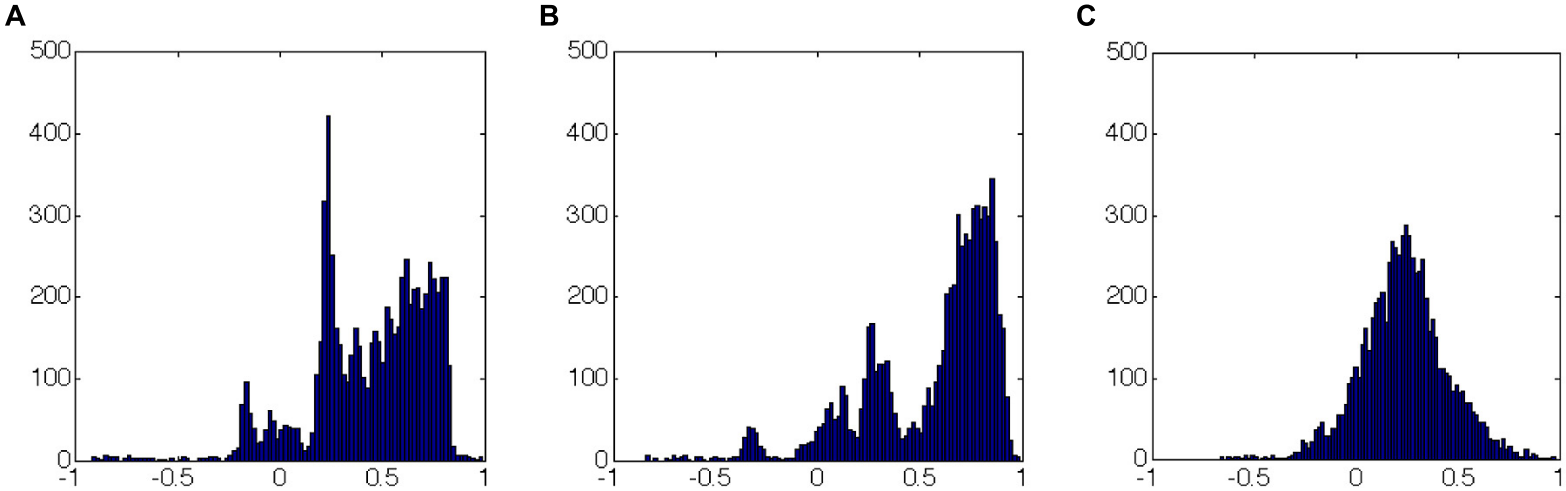
**Correlation coefficients distribution (over the whole time series of each experiment) as in Figure 5: (A,B) sleep; (C) dream activity.** It can be easily seen that the histograms have similar shapes (in terms of number and range of values) for the two similar rearing states (**A** and **B**, sleep). This picture does not allow to specify if the same links (correlation between features) have similar values.

**FIGURE 7:**
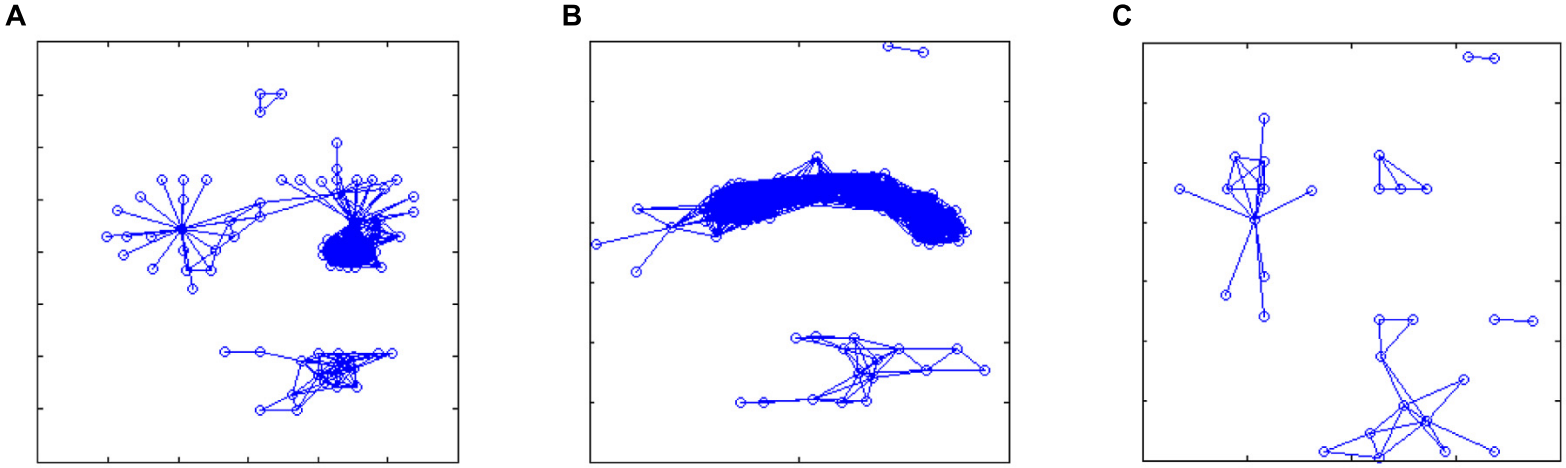
**Reconstructed networks in the three cases of Figure 5: (A,B) sleep; (C) dream activity.** Starting from the correlation matrices, an arbitrary threshold value was set (*r* > 0.8, but the results were qualitatively similar for a broader range of threshold values, from 0.75 to 0.85) in order to define significant links between features (expressing similarity over time of the linked features). These networks show which features are highly correlated during the different recordings, thus topological observables related to these network may provide a generalized representation of the different rearing states.

**FIGURE 8:**
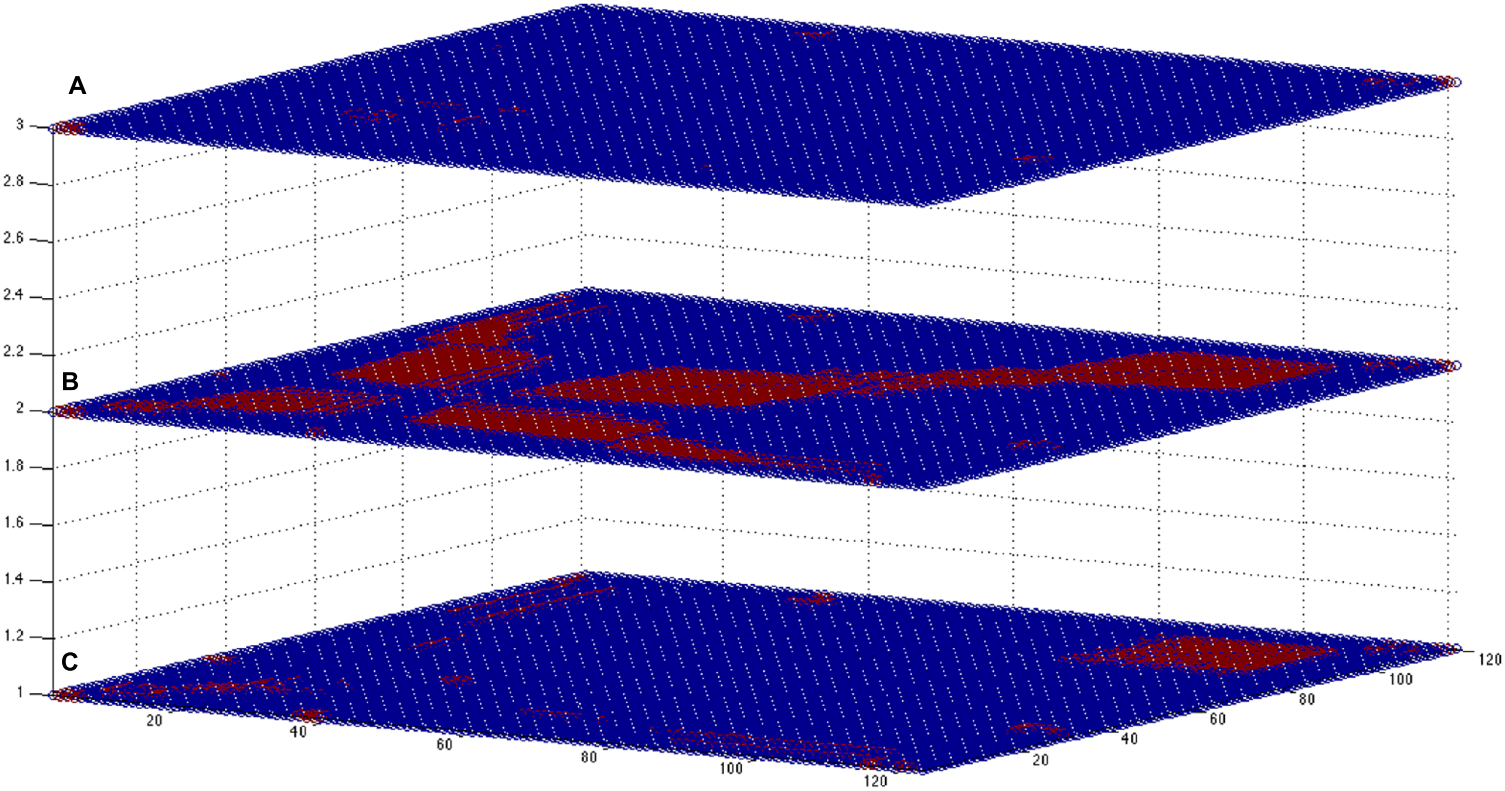
**Multiplex-like representation of correlation-based networks.** In the picture are shown the three square adjacency matrices (121 by 121, corresponding to the EEG extracted features) obtained for states (**A–C**; from bottom to top, respectively). Blue dots: no link; red dots, existing link. The overlap between the states is higher for cases **(A,B)**, expressing a similar brain state (corresponding to a sleep state): about 5.2% of the possible *N*(*N*- 1) links are the same for networks **(A)** and **(B)**, whereas for the other intersections the values are about 10 times smaller (0.3–0.5%). Adequate sampling statistics may help to define specific patterns characterizing each rearing state, and similarity measures can be performed to classify the different states.

As an example, here we apply the methods described previously in the cases of reconstruction of the gene expression data to experimental measurements obtained from the EEG device. As it can be seen (**Figure 5**), novel feature extraction methods can emphasize the differences and similarities between brain states. As a second step, a network reconstruction starting from time correlation of the selected features can be performed (**Figures 6** and **7**): the multiplex structure applied on the adjacency matrices in the three states (highlighting the links rather than the node structure of the network, **Figure 8**) allows to find which parts of the network are overlapping for the different states. An increasing number of recordings in different states, applied to different samples (in order to build a “compendium” of observations) will help in building a “library” onto which new experimental observations can be mapped.

## CONCLUSION

In our opinion, novel techniques (such as fNMR) and more classical techniques (such as EEG) must be integrated by novel processing and analysis tools, able to extract relevant features of the signal at the single-trace level, but also able to reveal significant interconnections (causal or associative) between traces. Moreover, any possible relevant biological information (e.g., about anatomic regions) must be integrated with the experimental data, in order to enrich the statistical significance of the performed analysis and its biological interpretation.

For these purposes, a great emphasis must be given to feature extraction methods (overcoming the classical Fourier analysis) and to network and multiplex approaches, that may allow to integrate the different informations both in time and space, and to take into account the global complexity of the signal. From this point of view, the panorama of analysis methods for brain data can be enormously enriched by the transfer of knowledge of already existing tools coming from the field of Systems Biology, which is exploiting network approaches and *a priori* biological knowledge since its beginning.

The pathway analysis and its generalization to networks and multiplexes gives the enormous possibility to merge in a unifying framework heterogeneous data as those arising from “omics” measurements and those arising from imaging and EEG. This possibility opens new scenarios for combining microscopic and macroscopic information on single patients that can shed new light in the field of personalized medicine.

## Conflict of Interest Statement

The authors declare that the research was conducted in the absence of any commercial or financial relationships that could be construed as a potential conflict of interest.

## GLOSSARY

### NETWORK

A network Newman (2003) is the schematical representation of a set of relationships (links) between elements (nodes). Mathematically it can be represented by a NxN square matrix (adjacency matrix, with N the number of nodes) with non-zero elements (equal to one for topological networks and to a real value for weighted networks) where a link exists between two nodes. Other representations are available, eg. a NxL incidence matrix (N number of nodes and L number of links) in which -1 and 1 values are put in each row corresponding to the leaving and the entering node. This formalism represents a sort of “generalized” derivative (or better a finite difference) for a function defined on the nodes, and is the basis for the Laplacian Operator formalism for networks.

### CENTRALITY

Measures for nodes, links or network subsets that help ranking these elements based on their topological/structural characteristics. Common centrality measures are connectivity degree (number of incoming/outgoing links), betweenness centrality (ratio of shortest paths passing through a node/link), eigenvalue centrality (like Google PageRank, in which a node is important if it is connected to important nodes, leading to an eigenvalue problem for the adjacency matrix). More recent measures, working in particular for dense and weighted networks, are salient links (Grady et al., 2012) and spectral centrality (Pauls and Remondini, 2012).

### MULTIPLEX

A multilayer network (multiplex) represents a set of networks in which the same nodes may appear onto different layers with different relationships. A multiplex can be thought for genes, which proteins appear in Transcription networks (as transcription factors), in Protein–Protein interaction networks (as proteins), and in Metabolic networks (as enzymes controlling metabolic reactions). In neuroscience, we can define a multiplex considering anatomical vs. functional networks, or neuronal networks characterized by different classes of neurotransmitters and receptors.

### COMMUNITIES

Networks very often can be dissected into parts, reflecting special relationships between nodes belonging to the same community. These groups can be defined by *a priori* knowledge (like different anatomical or functional regions) or deduced by network topological properties. Clustering methods can be applied to the network as a function of the chosen metrics (e.g., by paths or measures of overlap between node neighborhoods), or communities might arise from dynamical processes applied to the network (e.g., considering transient states of random walks over the network).

### NETWORK-BASED STATISTICS

More and more often Systems Biology is integrating common statistical tests (Student’s *T* test, ANOVA and their nonparametric variants) with null models derived from the network structure in which data are embedded. Single-probe statistics (for genes, proteins, neurons) can be scaled up to higher structures like biochemical pathways or brain regions in a recursive manner (Francesconi et al., 2008), and can be enriched by information about significance of their neighbourhood. Moreover, different network structures can be compared and a probability can be assigned to such comparisons in order to assess biological relevance of the observed structure (see a recent comment on Singleton, 2014).

## References

[B1] Affymetrix Inc.: Technical note: guide to probe logarithmic intensity error (PLIER) estimation. http://www.affymetrix.com/support/technical/technotesmain.affx.

[B2] Albert R. Barabási A.-L. (2002). Statistical mechanics of complex networks. *Rev. Mod. Phys.* 74 47 10.1103/RevModPhys.74.47

[B3] Ashburner M. Ball C. A. Blake J. A. Botstein D. Butler H. Cherry J. M. (2000). Gene ontology: tool for the unification of biology. The Gene Ontology Consortium. *Nat. Genet.* 25 25–29 10.1038/75556 10802651 PMC3037419

[B4] Beissbarth T. Speed T. P. (2004). GOstat: find statistically overrepresented Gene Ontologies within a group of genes. *Bioinformatics* 20 1464–1465 10.1093/bioinformatics/bth088 14962934

[B5] Bianconi G. (2013). Statistical mechanics of multiplex networks: entropy and overlap. *Phys. Rev. E Stat. Nonlin. Soft Matter Phys.* 87 062806 10.1103/PhysRevE.87.062806 23848728

[B6] Biederman I. (1987). Recognition-by-components: a theory of human image understanding. *Psychol. Rev.* 94 115 10.1037/0033-295X.94.2.115 3575582

[B7] Boccaletti S. Latora V. Moreno Y. Chavez M. Hwang D.-U. (2006). Complex networks: structure and dynamics. *Phys. Rep.* 424 175–308 10.1016/j.physrep.2005.10.009

[B8] Boorman S. A. Harrison C. (1976). White, social structure from multiple networks. II. Role structures. *Am. J. Sociol.* 81 1384–1446 10.1086/226228

[B9] Butte A. J. Kohane I. S. (1999). Unsupervised knowledge discovery in medical databases using relevance networks. *Proc. AMIA Symp.* 1999 711–715 10566452 PMC2232846

[B10] Buzsáki G. Draguhn A. (2004). Neuronal oscillations in cortical networks. *Science* 304 1926–1929 10.1126/science.1099745 15218136

[B11] Cabeza R. Nyberg L. (2000). Imaging cognition II: an empirical review of 275 PET and fMRI studies. *J. Cogn. Neurosci.* 12 1–47 10.1162/08989290051137585 10769304

[B12] Camacho D. de la Fuente A. Mendes P. (2005). The origin of correlations in metabolomics data. *Metabolomics* 1 53–63 10.1007/s11306-005-1107-3

[B13] Cai L. Friedman N. Xie X. S. (2006). Stochastic protein expression in individual cells at the single molecule level. *Nature* 440 358 10.1038/nature04599 16541077

[B14] Cooper L. N. Intrator N. Blais B. S. Shouva H. Z. (ed.). (2004). *Theory of Cortical Plasticity.* Singapore: World Scientific Publishing

[B15] Davidson R. J. (1988). EEG measures of cerebral asymmetry: conceptual and methodological issues. *Int. J. Neurosci.* 39 71–89 10.3109/00207458808985694 3290140

[B16] de la Fuente A. Bing N. Hoeschele I. Mendes P. (2004). Discovery of meaningful associations in genomic data using partial correlation coefficients. *Bioinformatics* 20 3565–3574 10.1093/bioinformatics/bth445 15284096

[B17] Delorme A. Makeig S. (2004). EEGLAB: an open source toolbox for analysis of single-trial EEG dynamics including independent component analysis. *J. Neurosci. Methods* 134 9–21 10.1016/j.jneumeth.2003.10.009 15102499

[B18] Dobra A. Hans C. Jones B. Nevins J. R. West M. (2004). Sparse graphical models for exploring gene expression data. *J. Multivar. Anal.* 90 196–212 10.1016/j.jmva.2004.02.009

[B19] Dorogovtsev S. N. Goltsev A. V. Mendes J. F. F. (2008). Critical phenomena in complex networks. *Rev. Mod. Phys.* 80 1275 10.1103/RevModPhys.80.1275

[B20] Duncan D. Talmon R. Zaveri H. P. Coifman R. R. (2013). Identifying preseizure state in intracranial EEG data using diffusion kernels. *Math. Biosci. Eng.* 10 579–590 10.3934/mbe.2013.10.579 23906137

[B21] Edelman S. (1999). *Representation and Recognition in Vision.* Cambridge: MIT Press

[B22] Fisher R. S. van Emde Boas W. Blume W. Elger C. Genton P. Lee P. (2005). Epileptic seizures and epilepsy: definitions proposed by the International League Against Epilepsy (ILAE) and the International Bureau for Epilepsy (IBE). *Epilepsia* 46 470–472 10.1111/j.0013-9580.2005.66104.x 15816939

[B23] Fortunel N. O. Otu H. H. Ng H. H. Chen J. Mu X. Chevassut T. (2003). Comment on ‘Stemness’: transcriptional profiling of embryonic and adult stem cells and a stem cell molecular signature. *Science* 302 393 10.1126/science.1086384 14563990

[B24] Francesconi M. Remondini D. Neretti N. Sedivy J. M. Cooper L. N. Verondini E. (2008). Reconstructing networks of pathways via significance analysis of their intersections. *BMC Bioinformatics* 9(Suppl. 4):S9 10.1186/1471-2105-9-S4-S9 PMC2367636 18460182

[B25] Friedman N. Cai L. Xie X. S. (2006). Linking stochastic dynamics to population distribution: an analytical framework of gene expression. *Phys. Rev. Lett.* 97 168302 10.1103/PhysRevLett.97.168302 17155441

[B26] Goeman J. J. van de Geer S. A. de Kort F. van Houwelingen H. C. (2004). A global test for groups of genes: testing association with a clinical outcome. *Bioinformatics* 20 93–99 10.1093/bioinformatics/btg382 14693814

[B27] Grady D. Thiemann C. Brockmann D. (2012). Robust classification of salient links in complex networks. *Nat. Commun.* 3 864 10.1038/ncomms1847 22643891

[B28] Granger C. W. J. (1988). Causality, cointegration, and control. *J. Econ. Dyn. Control* 12 551–559 10.1016/0165-1889(88)90055-3

[B29] Gross T. Blasius B. (2008). Adaptive coevolutionary networks: a review. *J. R. Soc. Interface* 5 259–271 10.1098/rsif.2007.1229 17971320 PMC2405905

[B30] Haas L. F. (2003). Hans Berger (1873–1941), Richard Caton (1842–1926), and electroencephalography. *J. Neurol. Neurosurg. Psychiatry* 74:9 10.1136/jnnp.74.1.9 PMC1738204 12486257

[B31] Haimovici A. Tagliazucchi E. Balenzuela P. Chialvo D. R. (2013). Brain organization into resting state networks emerges at criticality on a model of the human connectome. *Phys. Rev. Lett.* 110 178101 10.1103/PhysRevLett.110.178101 23679783

[B32] Hekstra D. Taussig A. R. Magnasco M. Naef F. (2003). Absolute mRNA concentrations from sequence-specific calibration of oligonucleotide arrays. *Nucleic Acids Res.* 31 1962–1968 10.1093/nar/gkg283 12655013 PMC152799

[B33] Henriques J. B. Davidson R. J. (1990). Regional brain electrical asymmetries discriminate between previously depressed and healthy control subjects. *J. Abnorm. Psychol.* 99 22–31 10.1037/0021-843X.99.1.22 2307762

[B34] Holme P. Newman M. E. J. (2006). Newman nonequilibrium phase transition in the coevolution of networks and opinions. *Phys. Rev. E Stat. Nonlin. Soft Matter Phys.* 74(Pt 2), 056108. 10.1103/PhysRevE.74.056108 17279969

[B35] Honey C. J. Kötter R. Breakspear M. Sporns O. (2007). Network structure of cerebral cortex shapes functional connectivity on multiple time scales. *Proc. Natl. Acad. Sci. U.S.A.* 104 10240–10245 10.1073/pnas.0701519104 17548818 PMC1891224

[B36] Irizarry R. A. Hobbs B. Collin F. Beazer-Barclay Y. D. Antonellis K. J. Scherf U. (2003). Exploration, normalization, and summaries of high density oligonucleotide array probe level data. *Biostatistics* 4 249–264 10.1093/biostatistics/4.2.249 12925520

[B37] Intrator N. (2014). *Brain Activity Features: A Continuous Window Into the Mind.* Preprint

[B38] Kandel E. R. Markram H. Matthews P. M. Yuste R. Koch C. (2013). Neuroscience thinks big (and collaboratively). *Nat. Rev. Neurosci.* 14 659–664 10.1038/nrn3578 23958663

[B39] Kanehisa M. Goto S. (2000). KEGG: kyoto encyclopedia of genes and genomes. *Nucleic Acids Res.* 28 27–30 10.1093/nar/28.1.27 10592173 PMC102409

[B40] Kishino H. Waddell P. J. (2000). Correspondence analysis of genes and tissue types and finding genetic links from microarray data. *Genome Inform. Ser. Workshop Genome Inform.* 11 83–95 11700590

[B41] Klimesch W. (1999). EEG alpha and theta oscillations reflect cognitive and memory performance: a review and analysis. *Brain Res. Rev.* 29 169–195 10.1016/S0165-0173(98)00056-3 10209231

[B42] Kurzweil R. Grossman T. (2005). *Fantastic Voyage: Live Long Enough to Live Forever.* London: Rodale 19745481

[B43] Latour B. (1987). *Science in Action: How to Follow Scientists and Engineers Through Society.* Milton Keynes: Open University Press

[B44] Law J. Hassard J. (eds). (1999). *Actor Network Theory and After.* Oxford and Keele: Blackwell and the Sociological Review

[B45] Liu X. Duyn J. H. (2013). Time-varying functional network information extracted from brief instances of spontaneous brain activity. *Proc. Natl. Acad. Sci. U.S.A.* 110 4392–4397 10.1073/pnas.1216856110 23440216 PMC3600481

[B46] Luck S. J. (2005). *An Introduction to the Event-Related Potential Technique (Cognitive Neuroscience)*. Cambridge: MIT Press

[B47] Lynch G. Kessler M. Arai A. Larson J. (1990). The nature and causes of hippocampal long-term potentiation. *Prog. Brain Res.* 83 233–250 10.1016/S0079-6123(08)61253-4 2168058

[B48] Magwene P. M. Kim J. (2004). Estimating genomic coexpression networks using first-order conditional independence. *Genome Biol.* 5 R100. 10.1186/gb-2004-5-12-r100 PMC545795 15575966

[B49] Manoli T. Gretz N. Gröne H. J. Kenzelmann M. Eils R. Brors B. (2006). Group testing for pathway analysis improves comparability of different microarray datasets. *Bioinformatics* 22 2500–2506 10.1093/bioinformatics/btl424 16895928

[B50] Martins A. M. Camacho D. Shuman J. Sha W. Mendes P. Shulaev V. (2004). A systems biology study of two distinct growth phases of *Saccharomyces cerevisiae* cultures. *Curr. Genomics* 5 649–663 10.2174/1389202043348643

[B51] Milanesi L. Romano P. Castellani G. Remondini D. Liò P. (2009). Trends in modeling biomedical complex systems. *BMC Bioinformatics* 10(Suppl. 12):I1 10.1186/1471-2105-10-S12-I1 PMC2762057 19828068

[B52] Mirowski P. Madhavan D. LeCun Y. Kuzniecky R. (2009). Classification of patterns of EEG synchronization for seizure prediction. *Clin. Neurophysiol.* 120 1927–1940 10.1016/j.clinph.2009.09.002 19837629

[B53] Mootha V. K. Lindgren C. M. Eriksson K. F. Subramanian A. Sihag S. Lehar J. (2003). PGC-1alpha-responsive genes involved in oxidative phosphorylation are coordinately downregulated in human diabetes. *Nat. Genet.* 34 267–273 10.1038/ng1180 12808457

[B54] Newman M. E. J. (2003). The structure and function of complex networks. *SIAM Rev.* 45 167–256 10.1137/S003614450342480

[B55] Niedermeyer E. Lopes da Silva F. H. (eds). (2005). *Electroencephalography: Basic Principles, Clinical Applications, and Related Fields*. Philadelphia, PA: Lippincott Williams & Wilkins

[B56] Pandey R. Guru R. K. Mount D. W. (2004). Pathway miner: extracting gene association networks from molecular pathways for predicting the biological significance of gene expression microarray data. *Bioinformatics* 20 2156–2158 10.1093/bioinformatics/bth215 15145817

[B57] Pauls S. D. Remondini D. (2012). Measures of centrality based on the spectrum of the Laplacian. *Phys. Rev. E Stat. Nonlin. Soft Matter Phys.* 85(Pt 2), 066127. 10.1103/PhysRevE.85.066127 23005182

[B58] Remondini D. O’Connell B. Intrator N. Sedivy J. M. Neretti N. Castellani G. C. (2005). Targeting c-Myc-activated genes with a correlation method: detection of global changes in large gene expression network dynamics. *Proc. Natl. Acad. Sci. U.S.A.* 102 6902–6906 10.1073/pnas.0502081102 15867157 PMC1100785

[B59] Schafer J. Strimmer K. (2005). An empirical Bayes approach to inferring large-scale gene association networks. *Bioinformatics* 21 754–764 10.1093/bioinformatics/bti062 15479708

[B60] Schreiber T. Schmitz A. (2000). Surrogate time series. *Phys. D* 142 346–382 10.1016/S0167-2789(00)00043-9

[B61] Singleton A. B. (2014). A unified process for neurological disease. *Science* 343 497–498 10.1126/science.1250172 24482474 PMC6478387

[B62] Subramanian A. Tamayo P. Mootha V. K. Mukherjee S. Ebert B. L. Gillette M. A. (2005). Gene set enrichment analysis: a knowledge-based approach for interpreting genome-wide expression profiles. *Proc. Natl. Acad. Sci. U.S.A.* 102 15545–15550 10.1073/pnas.0506580102 16199517 PMC1239896

[B63] Swartz B. E. (1998). The advantages of digital over analog recording techniques. *Electroencephalogr. Clin. Neurophysiol.* 106 113–117 10.1016/S0013-4694(97)00113-2 9741771

[B64] Szell M. Lambiotte R. Thurner S. (2010). Multirelational organization of large-scale social networks in an online world. *Proc. Natl. Acad. Sci. U.S.A.* 107 13636–13641 10.1073/pnas.1004008107 20643965 PMC2922277

[B65] Toh H. Horimoto K. (2002). Inference of a genetic network by a combined approach of cluster analysis and graphical Gaussian modeling. *Bioinformatics* 18 287–297 10.1093/bioinformatics/18.2.287 11847076

[B66] van Kampen N. G. (2007). *Stochastic Processes in Physics and Chemistry* 3rd edn. Amsterdam: North-Holland Personal Library

[B67] Waddell P. J. Kishino H. (2000). Cluster inference methods and graphical models evaluated on NCI60 microarray gene expression data. *Genome Inform. Ser. Workshop Genome Inform.* 11 129–140 11700594

[B68] Wasserman S. Faust K. (1994). *Social Network Analysis: Methods and Applications*. Cambridge: Cambridge University Press 10.1017/CBO9780511815478

[B69] Wille A. Zimmermann P. Vranova E. Furholz A. Laule O. Bleuler S. (2004). Sparse graphical Gaussian modeling of the isoprenoid gene network in *Arabidopsis thaliana*. *Genome Biol.* 5 R92. 10.1186/gb-2004-5-11-r92 PMC545783 15535868

[B70] Yang H. Long X. Y. Yang Y. Yan H. Zhu C. Z. Zhou X. P. (2007). Amplitude of low frequency fluctuation within visual areas revealed by resting-state functional MRI. *Neuroimage* 36 144–152 10.1016/j.neuroimage.2007.01.054 17434757

[B71] Yin J. Li H. (2012). Model selection and estimation in the matrix normal graphical model. *J. Multivar. Anal.* 107 119–140 10.1016/j.jmva.2012.01.005 22368309 PMC3285238

